# Nutritional Status After Total Gastrectomy for Gastric Cancer

**DOI:** 10.4021/wjon2010.04.196w

**Published:** 2010-04-30

**Authors:** Esther Una Cidon

**Affiliations:** Medical Oncology Service, Clinical University Hospital, C/ Ramon y Cajal s/n, 47005, Valladolid, Spain. Email: aunacid@hotmail.com

**Keywords:** Gastrectomy, Gastric cancer, Nutritional Status

## Abstract

**Background:**

Gastric cancer is one of the most frequent causes of death secondary to cancer in the world. Surgery is the only potentially curative treatment but its clinical consequences are significant. The objective of this study is to evaluate the nutritional state of patients with a total gastrectomy secondary to gastric adenocarcinoma.

**Methods:**

We designed a descriptive study with a transversal cut in our institution. We included 22 patients which had a minimum evolution time of six months after total gastrectomy secondary to gastric cancer surgery was performed. Neither of them had metastasis. The nutritional analysis included only biochemical data. Descriptive statistics were used for statistical analysis.

**Results:**

Eight females and 14 males were included in the study. Median age was 57 years (34 - 69 years). The 74% of the patients were underweight and none of them was overweight. The average body mass index (BMI) was 16.88 kg/m^2^. Eleven patients suffered from mild anemia (10.5 - 12 g/dl) and 5 from moderate anemia (9 - 10.5 g/dl). Only two patients presented severe anemia (less than 9 g/dl). The 58% presented hypoproteinaemia and hypoalbuminaemia. The main post-surgery complication was nausea (46%). Seventy-eight percent of the patients had loss of appetite. Twenty-one patients were able to walk without help and leave their homes.

**Conclusions:**

The incidence of anemia in these patients was very high. In most of the patients, albumin and proteins levels were affected too. So malnutrition was a relevant consequence of a total gastrectomy.

## Introduction

Although the incidence of gastric cancer has been decreasing in last years, it continues being the second cause of cancer related mortality in the world [1]. In these patients, radical surgery with the aim of removal the complete gastric lesion with clear margins and its potentially involved regional lymph nodes is the only potentially curative treatment [2, 3].

This surgical procedure is called gastrectomy and consists of a surgical approach with the aim of partial or full surgical removal of the stomach followed always by a reconstruction of digestive transit [4]. Although it is also used to treat benign diseases such as perforations, it is most frequently applied in the treatment of malignant gastric diseases such as gastric adenocarcinoma. But the adverse effects of this type of surgery are significant, including a wide range of digestive symptoms such as loss of weight, anorexia and nausea among others and these should be balanced with the expected benefits in survival [5].

A lot has been discussed about the better surgical digestive transit reconstructive procedure to allow the correct oral intake and avoid malnutrition, however, direct termino-lateral esophagojejunostomy with preservation of the duodenal passage or Roux-en-Y jejunostomy might be considered the standard procedures on reconstruction [6] in several institutions. Despite all these techniques, incapacitating nutritional debilitation has often been regarded as a necessary consequence of the operative procedure [5].

All patients underwent a gastrectomy will suffer from weight loss probably because of the secondary alterations in the physiology of digestion and absorption induced by the surgical procedure per se [7, 8]. But not only weight loss is seen in these patients. Accompanying this symptom, we can also observe postoperative malnutrition with controversial causes. Many authors have advocated malabsorption as the main origin but others have suggested the inadequate caloric intake as the main problem [5].

Based upon the latter premise, it has been proposed that the construction of gastric pouches would be relevant in order to increase postoperative food intake regardless of the procedures evidence of malnutrition that has been described [9, 10].

Although the final cause of postgastrectomy malnutrition has not been clearly determined, it is evident that the mechanisms behind malnutrition are multifactorial and finally will deteriorate the quality of life of these patients [5]. At this point it becomes very important to treat these patients very early with a multidisciplinary approach including a nutritionist and involving nurses and primary health care in the nutritional education of these patients.

To know the nutritional status of patients underwent total gastrectomy secondary to gastric cancer carried out in our University Hospital, we designed a study with this objective.

## Patients and Methods

We designed a descriptive study with a transversal cut in our institution. This study was conducted from June 2006 to September 2008. We reviewed the medical records of 74 patients diagnosed with gastric cancer collected from the clinical registries of our hospital. We considered for our study 22 eligible patients who met the following criteria: all eligible patients had undergone a total gastrectomy and an esophago-jejunostomy Roux-en-Y as a reconstructive procedure at the General Surgery Department in our University Hospital. We only considered patients who had a minimum evolution time of six months after the surgical procedure was performed and a maximum of nine months and all data collected were referred to this period of time. Patients receiving chemotherapy after surgery or radiotherapy were excluded.

Patients with suspected or confirmed recurrence or metastases of cancer were excluded as well. All patients were on regularly follow-up after surgery every three months and all of them had a complete haematological and biochemical count and were available for the collection of anthropometric data.

For the nutritional assessments that were used: a) biochemical data such as proteinemia and albuminemia; b) anthropometric data such as weight, height, body mass index (BMI), we evaluated also late postoperative gastrointestinal symptoms. We applied descriptive statistics for statistical analysis of our data. The study was approved by the Hospital Research Committee.

## Results

The patients included were 8 (37%) females and 14 (63%) males (Table 1) with a median age of 57 years (range, 34 - 69 years). All patients have undergone total gastrectomy indicated by gastric cancer. None of the patients included had biochemical or anthropometric parameters suggesting malnutrition previous to diagnosis of gastric cancer and none of them presented significant weight loss before surgery (considering this point as the loss of weight more than 10% of the basal weight in the six months before surgery).

**Table 1:** Basal Characteristics of the Population

Total Number of Patients	22
Sex	
Females	8
Males	14
Median Age (range)	57 (34 - 69)
Stage (TNM classification)	
I	7
II	13
III	2
Histologic Subtype	
Diffuse signet-ring cells	7
Intestinal	15

The 74% of the patients were underweight and none of them was overweight. The average body mass index (BMI) was 16.88 kg/m^2^ (± 2.5 kg/ m^2^).

The 50% of the patients suffered from mild anemia (with a range between 10.5 - 12 g/dl) and five patients (22.7%) presented moderate anemia (between 9 - 10.5 g/dl). Only two patients presented severe anemia (less than 9 g/dl) and four of them had a hematologic count within normal limits. The entire study population presented analytical ferropenia but all patients had normal levels of B_12_ vitamin because they had subjected to chronic supplementation after surgery.

The main immediate post-surgery complication was nausea which affected to 46% of the patients. But the most frequent symptom in our population was loss of appetite which affected to 78% of the patients. The number of meals was three to six, with an average of five per day. Other symptoms were nonspecific abdominal complaints and epigastric fullness. Just one patient presented late dumping and 34% had a negative attitude towards food. Early dumping was presented by six patients (27%) but it was completely solved in months in five of them.

We have not detected diarrhoea or abdominal pain. Nonspecific abdominal discomfort was referred by 14 patients.

These symptoms not only affected the normal daily live of these patients but prevented them from making adequate caloric intake. The 58% presented hypoproteinaemia and hypoalbuminaemia (less than 3.5 g/dl).

Twenty-one patients out of 22 were able to walk without help and leave their homes. But all of them presented fatigue and adynamia.

Dairy products were the food poorly tolerated by these patients with the low intake of calcium as a main consequence.

## Discussion

Gastric cancer continues being the second cause of cancer-related death worldwide and gastrectomy remains the standard treatment procedure with a potentially curative aim [1, 2]. Although it is now more than one century since the first successful removal of a gastric neoplasm and the surgical reconstructive techniques and anesthetics methods have widely improved, the operative procedure continues having many gastrointestinal complications with impact on quality of life of these patients. Thus the improvement obtained in these surgical procedures is far from complete.

Controversies still exist regarding the best methods to ensure the reconstruction and continuity of gastrointestinal transit after a total gastrectomy has been performed [11].

All reconstructive procedures aim to achieve the complete and fast recovery of oral intake in patients subjected to a potentially curative treatment for gastric cancer and who might need to complete their treatment with adjuvant aggressive therapies such as chemotherapy and radiotherapy or simply to quickly return to their normal life.

Kalmar K. published an article [12] presenting the results after comparing several reconstructive methods following total gastrectomy. Three prospective studies and a clinical experiment were carried out to evaluate postoperative weight, BMI, nutritional and immunological laboratory parameters, gut motility, lipid and carbohydrate absorption, quality of life, and gastrointestinal hormone production.

The first trial compared Roux-en-Y to an aboral pouch construction and the second study compared these two methods and a third one with aboral pouch construction but with a preserved duodenal passage. These studies concluded that lipid absorption and quality of life were better in the patients underwent the aboral pouch construction. In addition to these facts, in the cases with preserved duodenal passage the iron metabolism was better too. With the third trial which compared aboral pouch to conventional oral pouch, both methods with duodenal passage preserving versions, they did not observed differences. In the clinical experiment where they measured hormonal levels, they concluded that duodenal passage preserving reconstruction provided the most physiological levels.

Espat N.J. and Karpeh M. [11] have reviewed the different methods to reconstruct digestive transit and conclude that Roux-en-Y esophago-jejunostomy with or without a pouch reservoir is the most frequently used procedure after total gastrectomy. Others preferred preserving duodenal passage alluding that it is more physiological surgery.

It is clear that a defined optimal method to reconstruct digestive transit has not been established yet. Thus in our institution, esophago-jejunostomy Roux-en-Y is considered the standard procedure.

Despite all these methods, many patients continue to present analytical parameters or anthropometric malnutrition that varies according to the clinical series published.

In our study we have obtained similar results to the Delgado del Rey et al study [13]. They have included 45 patients who underwent a total gastrectomy and they refer the collected data to their first visit following surgery although they subdivided the patients in three groups depending on the time that had passed since the gastrectomy had been performed (less than three months, from three months to a year and over one year) and they concluded that there was no significant differences between these groups in any of the parameters (clinical and analytical) evaluated.

These results suggest that the changes after gastrectomy are maintained over the time.

In this study the most frequent complication after surgery was diarrhoea affecting to 31% of the patients, followed by pain and early dumping. By the contrary in our population, we observed that loss of appetite and nausea were the most relevant and frequent symptoms. We have not detected cases with diarrhoea or pain. Instead, we observed abdominal discomfort difficult to explain. Our study obtained lower rates of dumping syndrome both early and late and similar proportion of patients with negative attitudes towards food.

The Worldwide Health Organization has established that a BMI less than 18.5 kg/m^2^ means malnutrition. Our study population showed a BMI lower than this as an average indicating the high rate of malnutrition in this population. Other studies have shown BMI higher than ours, most of them more than 20 kg/m^2^, although many of these studies have included patients with partial gastrectomy and consequently better nutritional parameters.

Although we observed high rates of anemia, mild in the majority of the cases, this analytical alteration did not noticeably affect the normal life of our patients although all of them presented mild adynamia.

Despite all these complications, just one patient was unable to walk independently. In the study referred before they obtained a high proportion of patients very affected with impossibility to leave home (the 43%) and only 13% were able to work.

Regardless of the differences between several studies, we can conclude that it is necessary to improve clinical and analytical nutritional parameters in these patients. To achieve this goal, it is necessary to use a multidisciplinary approach early after surgery that should include also a nutritionist to improve global situation of these patients.

## References

[1] Cruz Hernandez JJ (2004). Lecciones de Oncologia Clinica.

[2] Allum WH, Powell DJ, McConkey CC, Fielding JW (1989). Gastric cancer: a 25-year review. Br J Surg.

[3] Lundh G, Burn JI, Kolig G, Richard CA, Thomson JW, van Elk PJ, Oszacki J (1974). A co-operative international study of gastric cancer (under the auspices of the International Federation of Surgical Colleges). Ann R Coll Surg Engl.

[4] Fly OA, Priestley JT, Comfort MW, Gage RP (1958). Total gastrectomy: mortality and survival. Ann Surg.

[5] Bradley EL, Isaacs J, Hersh T, Davidson ED, Millikan W (1975). Nutritional consequences of total gastrectomy. Ann Surg.

[6] Blochle C, Mann O, Stenger AM, Busch C, Izbicki JR (2001). [Gastric substitute following gastrectomy]. Zentralbl Chir.

[7] Brain RH, Stammers FA (1951). Sequelae of radical gastric resections. Clinical and metabolic findings in 35 cases. Lancet.

[8] Macdonald RM, Ingelfinger FJ, Belding HW (1947). Late effects of total gastrectomy in man. N Engl J Med.

[9] Moreno AH (1956). Studies on nutritional and other disturbances following operations for cancer of the stomach; with particular reference to the use of a jejunal pouch as a substitute gastric reservoir. Ann Surg.

[10] Knox WG (1965). Use of a Jejunal Pouch in Treating Post-Gastrectomy Malnutrition. Ann Surg.

[11] Espat NJ, Karpeh M (1998). Reconstruction following total gastrectomy: a review and summary of the randomized prospective clinical trials. Surg Oncol.

[12] Kalmar K (2008). [Searching for the optimal reconstructive methods following total gastrectomy]. Magy Seb.

[13] Delgado del Rey M, Gomez Candela C, Cos Blanco AI, Iglesias Rosado C, Fernandez Ibanez MV, Castillo Rabaneda R, Mateo Lobo R (2002). [Nutritional evaluation in patients with total gastrectomy]. Nutr Hosp.

